# Elucidating the mechanisms underlying differential anthocyanin biosynthesis and its link to stem color and root isoflavonoid levels in *Astragalus membranaceus* var. *mongholicus*

**DOI:** 10.1093/hr/uhag088

**Published:** 2026-03-04

**Authors:** Yi Chen, Sifei Duan, Meng Zhang, Yang-oujie Bao, Yungang Tian, Xuehui Dong, Min Ye

**Affiliations:** State Key Laboratory of Natural and Biomimetic Drugs, School of Pharmaceutical Sciences, Peking University, Beijing 100191, China; College of Agronomy and Biotechnology, China Agricultural University, Beijing 100193, China; State Key Laboratory of Natural and Biomimetic Drugs, School of Pharmaceutical Sciences, Peking University, Beijing 100191, China; State Key Laboratory of Natural and Biomimetic Drugs, School of Pharmaceutical Sciences, Peking University, Beijing 100191, China; State Key Laboratory of Natural and Biomimetic Drugs, School of Pharmaceutical Sciences, Peking University, Beijing 100191, China; College of Agronomy and Biotechnology, China Agricultural University, Beijing 100193, China; State Key Laboratory of Natural and Biomimetic Drugs, School of Pharmaceutical Sciences, Peking University, Beijing 100191, China

## Abstract

*Astragalus membranaceus* var. *mongholicus* (AMM) is the principal botanical source of Huangqi, a traditional medicinal herb whose therapeutic value primarily stems from the accumulation of isoflavones and other bioactive compounds in the roots. In this study, field surveys across major AMM production regions revealed pronounced natural variation in stem coloration. Chemical analysis showed that the roots of the red-stemmed type contained significantly higher levels of four bioactive isoflavones and volatile organic compounds than those in green-stemmed plants. Metabolomic profiling further revealed a specific enrichment of cyanidin-based anthocyanins in the red stems, establishing the metabolic basis of the red stem phenotype. Both transcriptomic and metabolomic analyses indicated an overall upregulation of the flavonoid and phenylpropanoid biosynthetic pathways in the stem and root tissues of red-stemmed AMM. Weighted gene co-expression network analysis (WGCNA) identified six key genes (*AmC4H*, *AmCHS*, *AmCHI*, *AmF3H*, *AmF3*′*H*, and *AmBZ1*) that were strongly associated with the red stem phenotype, all of which were specifically highly expressed in red stems. Functional assays confirmed their roles in anthocyanin biosynthesis. Molecular modeling provided further insights into the substrate specificity of AmBZ1. This study proposes stem color as a visible phenotypic reference for early-stage germplasm selection in AMM, and characterizes the molecular basis underlying red stem formation, providing a foundation for elite germplasm development and molecular breeding.

## Introduction

*Astragalus membranaceus* var. *mongholicus* (AMM) is the principal botanical source of Huangqi, a medicinal herb that holds a prominent position in the Chinese traditional medicine (TCM) system [1, 2]. Its medicinal use in China dates back to at least 1800 years ago of Han Dynasty [3]. Modern pharmacological studies have demonstrated that Huangqi possesses a wide range of therapeutic properties, including immunomodulatory [4, 5] anti-fatigue [6, 7], hypoglycemic [8], antioxidant [9], and anti-tumor activities [10, 11]. In addition to its medicinal applications, AMM is also widely used in functional food and livestock industries [12, 13]. Owing to the high nutritional value, the aerial parts of the plant are often used as a source of high-quality forage.

The pharmacological activity of AMM is primarily attributed to the accumulation of bioactive compounds in its roots, including flavonoids, saponins, and polysaccharides. Among them, the biosynthetic pathways of key compounds such as calycosin-7-*O*-*β*-d-glucoside and astragaloside IV have been elucidated [14–17], providing a theoretical foundation for further investigation into their regulatory mechanisms and the advancement of molecular breeding. However, the biosynthesis and accumulation of these compounds are influenced by genotype, environment, and their interactions [18]. As an integrated outcome of both genetic and environmental factors, plant phenotypes often exhibit variability associated with changes in the composition and levels of secondary metabolites, thereby affecting the quality and application potential of medicinal materials [19].

**Figure 1:**
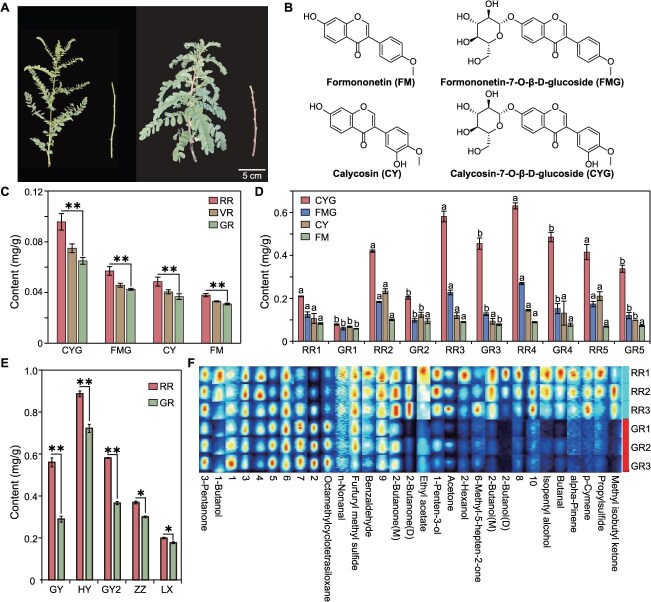
Phenotypic and chemical differences between red-stemmed and green-stinsufficient to reprogram the enzymeemmed AMM. (A) Characteristics of above-ground parts. (B) Structures of four isoflavones including formononetin, calycosin, formononetin 7-*O*-*β*-d-glucoside, and calycosin 7-*O*-*β*-d-glucoside. (C) Contents of four representative isoflavones in RR, VR, and GR of 5-month-old AMM plants cultivated under identical greenhouse conditions. Unpaired two-tailed *t*-test, *P* < 0.05, ^**^P < 0.01. (D) Contents of four representative isoflavones in GR and RR roots of plants aged 1 to 5 years. Means ± SD; *n* = 3 biological replicates. Different lowercase letters indicate statistically significant differences (*P* < 0.05) in the content of the four isoflavones among different year-old GR and RR samples, as determined by one-way ANOVA (E) Total contents of the four isoflavones in GR and RR roots collected from five different production regions. GY, Guyang County, Inner Mongolia Autonomous Region; HY, Hunyuan County, Shanxi Province; GY2, Guyuan County, Ningxia Hui Autonomous Region; ZZ, Zizhou County, Shaanxi Province; NX, Longxi County, Gansu Province. (F) Root VOC fingerprint profiles of GR and RR samples. The structures of the VOCs are provided in Fig. S1.

Based on this premise, we conducted field surveys in two major production regions—Hunyuan County in Shanxi Province and Zizhou County in Shaanxi Province. Across both sites, nine phenotypic traits were recorded, all of which showed varying degrees of natural variation (Table 1). Notably, stem color exhibited particularly high variability at both sites (Fig. 1A), with coefficients of variation reaching 44.05% and 44.75%, respectively, indicating substantial differentiation within the cultivated populations. To further evaluate the prevalence of this trait in natural populations, we conducted a supplementary survey of stem coloration in 873 wild AMM accessions from 47 natural populations across 8 provinces, all of which are conserved in our germplasm repository (Table S1). The results showed that stem color exhibited a high coefficient of variation within these natural populations (Table S2). However, systematic studies on root quality differences among AMM accessions with different stem colors are still lacking. Therefore, to elucidate the relationship between stem color and the accumulation of root bioactive compounds is crucial for precision breeding and standardized cultivation of AMM, with the goal of ensuring herb drug quality consistency.

**Table 1:** Summary of phenotypic traits of AMM in two major production regions.

Trait	Production region	Minimum value	Maximum value	Mean	Standard deviation	Coefficient of variation (%)
Plant height (cm)	Hunyuan	35	110	79.12	15.72	19.87
	Zizhou	55	120	84.27	15.65	18.57
Number of leaflets	Hunyuan	19	37	27.41	3.88	14.16
	Zizhou	23	39	29.39	3.58	12.190
Number of main stem branches	Hunyuan	6	21	12.54	3.20	25.53
	Zizhou	7	31	13.69	4.81	35.10
Number of stems per plant	Hunyuan	1	17	6.03	3.65	60.46
	Zizhou	1	14	5.05	3.80	75.28
Stem color	Hunyuan	1	3	1.30	0.60	44.05
	Zizhou	1	3	1.34	0.60	44.75
Plant type	Hunyuan	1	3	1.89	0.43	23.47
	Zizhou	1	3	1.90	0.350	18.32
Leaflet shape	Hunyuan	1	4	2.30	0.60	26.28
	Zizhou	1	3	2.37	0.55	23.21
Pod shape	Hunyuan	1	2	1.39	0.49	35.25
	Zizhou	1	2	1.39	0.49	35.40
Pod color	Hunyuan	1	5	2.37	1.21	51.07
	Zizhou	1	4	2.23	0.78	34.91

Phenotype codes: stem color: 1 (green), 2 (variegated), 3 (red); plant type: 1 (prostrate), 2 (semi-erect), 3 (erect); leaflet shape: 1 (orbicular), 2 (ovate), 3 (elliptic), 4 (oblong); pod shape: 1 (semi-ovate), 2 (semi-elliptic); pod color: 1 (light green), 2 (green with red), 3 (dark green), 4 (red).

Plant color traits are typically associated with the accumulation of pigments, such as anthocyanins, betalains, and carotenoids [20]. Previous studies have demonstrated that the diversity of petal coloration in roses is largely governed by genetic regulation of the anthocyanin biosynthetic pathway [21, 22], while fruit color variation in tomato (*Solanum lycopersicum*) is linked to the accumulation of carotenoids and other nutritional compounds [23]. In most angiosperms that do not produce betalains, red, purple, and blue hues are primarily determined by anthocyanins [24]. Anthocyanins are a class of flavonoid secondary metabolites synthesized via the phenylpropanoid pathway. The biosynthesis begins with the conversion of phenylalanine through PAL, C4H, and 4CL enzymes, followed by the formation of the flavanone backbone via CHS and CHI. Subsequent hydroxylation by F3H and F3′H/F3′5′H leads to the production of dihydroflavonols, which are then converted into anthocyanidin precursors by DFR and ANS. These compounds are finally stabilized through glycosylation, primarily catalyzed by BZ1, resulting in the formation of stable anthocyanins [20]. At the regulatory level, this pathway is mainly controlled by the MYB–bHLH–WD40 transcriptional complex and further modulated by various transporter proteins, such as GSTs, MATEs, and ABC transporters, as well as vacuolar sequestration mechanisms [25–27]. These regulatory layers collectively determine the spatial and temporal distribution of pigments within plant tissues. Although the anthocyanin biosynthetic pathway and its regulatory mechanisms have been reported in *Arabidopsis thaliana* and other plant species, the metabolic and molecular mechanisms underlying stem color variation in AMM remain largely unexplored.

This study aims to systematically compare root quality among AMM accessions with different stem colors. By integrating metabolomic and transcriptomic analyses, we investigate the metabolic features and molecular mechanisms underlying the red stem phenotype. Furthermore, *in vivo* and *in vitro* functional assays are conducted to validate the roles of key genes involved in stem color formation. The outcomes of this study will offer theoretical insights into the molecular mechanisms governing quality formation in AMM and provide a foundation for rational utilization and genetic improvement of its germplasm resources.

## Results

### Characterization of isoflavone composition in the roots of AMM with different stem colors

Isoflavones are key indicators for quality control of AMM. The Chinese Pharmacopoeia specifies calycosin 7-*O*-*β*-d-glucoside for the quality control of Huangqi, whereas the US Pharmacopoeia stipulates a set of four compounds comprising calycosin 7-*O*-*β*-d-glucoside, calycosin, formononetin 7-*O*-*β*-d-glucoside, and formononetin (Fig. 1B). To investigate whether stem color is associated with root isoflavone content, we first analyzed 5-month-old red-stemmed roots (RR), variegated-stemmed roots (VR), and green-stemmed roots (GR) AMM plants cultivated under identical greenhouse conditions. All four isoflavones were quantified using high-performance liquid chromatography with ultraviolet detection (HPLC-UV). RR samples exhibited the highest compound levels, while GR showed the lowest. Notably, VR samples displayed intermediate accumulation, suggesting that stem pigmentation is linked to a metabolic gradient in root isoflavone levels (Fig. 1C).

We next collected and analyzed RR and GR samples from field-grown AMM plants aged 1–5 years (Fig. 1D). The results showed that isoflavone contents increased progressively with plant age and reached a peak in the fourth year. In plants of the same age, the RR consistently contained significantly higher levels of all the four isoflavones compared to GR. For example, in 2-year-old plants, the content of calycosin 7-*O*-*β*-d-glucoside in RR reached 0.42 mg/g dry weight (DW), whereas that in GR was only 0.20 mg/g DW. Additionally, root samples from AMM accessions with different stem colors, collected from five production regions, were analyzed for further comparison. Quantitative analysis revealed that the total content of the four isoflavones was significantly higher in RR than in GR across all locations (Fig. 1E). Specifically, in the FS region, the total isoflavone content reached 0.56 mg/g DW in RR and 0.29 mg/g DW in GR. These results indicate that stem color, as a visible morphological trait, shows a consistent correlation with the accumulation of major root isoflavones across developmental stages and geographic regions. This association suggests that stem coloration may provide a useful phenotypic reference for estimating phytochemical variation in AMM under comparable genetic backgrounds.

### Comparative analysis of VOCs in roots of AMM with different stem colors

Volatile organic compounds (VOCs) are important reference indicators for the quality evaluation of Huangqi. Therefore, a strong bean-like aroma is considered a mark of superior quality [28]. In this study, gas chromatography-ion mobility spectrometry (GC-IMS) was used to compare the volatile metabolite profiles in the roots of AMM with different stem colors (Fig. S2). The difference comparison model revealed that the overall abundance of VOCs was higher in RR than in GR (Fig. S3). Further analysis using ion mobility spectrometry-based fingerprinting (Fig. 1F; Table S3) revealed that multiple volatile compounds such as methyl isobutyl ketone, *p*-cymene, butanal, isopentyl alcohol, 2-butanol (M), acetone, 1-penten-3-ol, 2-butanone (D), 2-butanone (M), and benzaldehyde were significantly more abundant in RR compared to GR. These results indicate that AMM roots with different stem colors exhibit systematic differences in both the composition and abundance of VOCs.

### Identification of characteristic metabolites associated with the red stem phenotype

**Figure 2:**
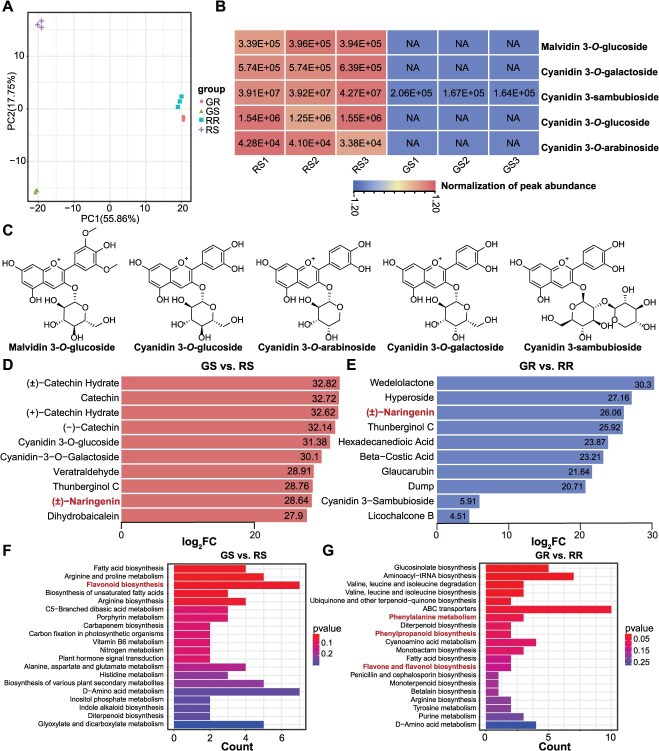
Metabolic basis of stem color variation in AMM. (A) PCA of metabolic profiles in RS, GS, RR, and GR samples. (B) Relative contents of five anthocyanins in RS and GS. Numbers correspond to peak abundance values. (C) Chemical structures of five anthocyanins. (D) and (E) Top 10 upregulated DAMs in GS vs. RS and GR vs. RR comparison groups. (F) and (G) KEGG enrichment analyses of DAMs identified in GS vs. RS and GR vs. RR comparisons.

To compare metabolic differences between root and stem tissues in AMM plants with different stem colors, a widely targeted metabolomics approach was applied. In total, 784 metabolites were identified, including 110 terpenoids, 82 flavonoids, 14 phenylpropanoids, and 5 coumarins (Table S4). After data normalization, we conducted principal component analysis (PCA) (Fig. 2A), orthogonal partial least squares discriminant analysis (OPLS-DA) (Fig. S4), and Spearman correlation analysis (Fig. S5). The results showed high within-group consistency and clear between-group separation, indicating reliable data quality and substantial differences in metabolic composition between root and stem tissues of different stem color types.

To further investigate the metabolic basis of red stem formation, differential accumulation metabolites (DAMs) were screened between green-stemmed stems (GS) and red-stemmed stems (RS) using a dual-threshold criterion of variable importance in projection (VIP) > 1.0 and fold change (FC) > 2 or < 0.5 (Figs S6 and S7; Tables S5 and S6). Among the differentially accumulated compounds, five anthocyanins, including malvidin 3-*O*-glucoside, cyanidin 3-*O*-glucoside, cyanidin 3-*O*-galactoside, cyanidin 3-*O*-arabinoside, and cyanidin 3-sambubioside were specifically enriched in RS (Fig. 2B and C). Notably, the first four anthocyanins were not detected in GR, and the abundance of cyanidin 3-sambubioside in RS was 225 times higher than in GS. These findings suggest that these anthocyanins likely play a key role in red stem development and that the cyanidin branch of anthocyanin biosynthesis may represent a critical metabolic pathway contributing to the red stem phenotype.

### Influence of stem color on root and stem metabolomic profiles in AMM

Based on the FC ranking, the top 10 upregulated DAMs were identified in both the GS vs. RS and GR vs. RR comparison groups (Fig. 2D-E). Notably, (±)-naringenin and thunberginol C were commonly enriched among the significantly upregulated metabolites in both comparisons. (±)-Naringenin is a key flavanone intermediate in the flavonoid biosynthesis pathway. KEGG enrichment analysis further revealed that the differential metabolites were predominantly enriched in secondary metabolic pathways, including flavonoid biosynthesis and phenylalanine metabolism (Fig. 2F and G). These results suggest that stem coloration may broadly influence the synthesis and accumulation patterns of flavonoid metabolites in both stem and root tissues of AMM.

### Identification of candidate genes associated with red stem formation

To investigate gene expression differences between stem and root tissues within AMM accessions exhibiting distinct stem pigmentation, we performed transcriptome sequencing on four tissue types, including RS, GS, RR, and GR tissues. PCA revealed clear separation among the four groups (Fig. S8), indicating distinct gene expression profiles across stem color types. Differential expression analysis and KEGG enrichment were conducted for both GS vs. RS and GR vs. RR comparisons (Fig. S9, Tables S7 and S8). A total of 1018 and 386 differentially expressed genes (DEGs) were identified in the GS vs. RS and GR vs. RR comparisons, respectively, using a threshold of fold change (FC) ≥ 2 and false discovery rate (FDR) < 0.05. The results showed that DEGs in both comparisons were significantly enriched in pathways related to flavonoid biosynthesis and phenylalanine metabolism. These findings are consistent with the metabolomic results, together supporting the association between stem coloration and transcriptional shifts in flavonoid metabolism in both stem and root tissues of AMM.

**Figure 3:**
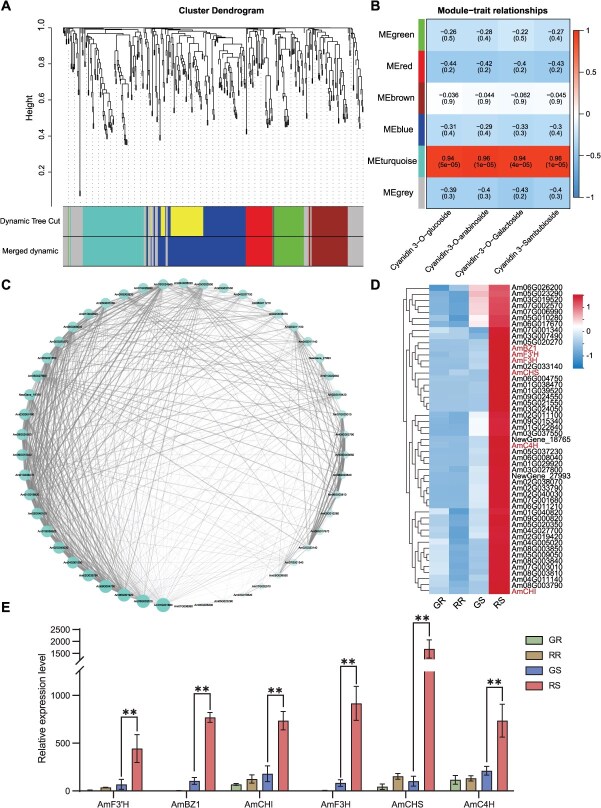
Identification of candidate genes associated with red stem formation in AMM. (A) Hierarchical clustering tree of co-expression modules identified by WGCNA. (B) Correlation between modules and anthocyanins. (C) Co-expression network of hub genes in the turquoise module. The larger the circle, the more edges. (D) Heatmap of hub genes in the turquoise module. (E) qRT-PCR shows expression levels of six key genes in the anthocyanin biosynthesis pathway in GS, RS, GR, and RR. ^*^P < 0.05, ^**^P < 0.01.

To further dissect gene modules associated with the red stem phenotype, Weighted gene co-expression network analysis (WGCNA) was conducted based on DEGs between GS and RS. A total of six co-expression modules were identified (Fig. 3A, Table S9). Correlation analysis between modules and anthocyanin metabolites showed that the turquoise module was highly positively correlated with cyanidin 3-*O*-glucoside, cyanidin 3-*O*-arabinoside, cyanidin 3-*O*-galactoside, and cyanidin 3-sambubioside (*r*^2^ > 0.94, *P* < 5 × 10^−5^) (Fig. 3B), suggesting its potential role in regulating the red stem trait. Based on high module membership (kME ≥ 0.8), 51 hub genes were identified from the turquoise module (Fig. 3C, Table S10), all of which exhibited markedly higher expression in RS tissues compared to other samples (Fig. 3D). Among these genes, *AmF3*′*H* (Am03G033510), *AmBZ1* (Am06G001920), *AmCHI* (Am02G040100), *AmF3H* (Am01G019920), *AmCHS* (Am04G001550), and *AmC4H* (Am03G024940) were identified as being involved in anthocyanin biosynthesis. Co-expression network construction using Cytoscape further revealed that *AmF3*′*H* and *AmBZ1* exhibited the highest connectivity within the module, with 42 and 41 edges, respectively (Fig. 3C), suggesting their potential roles as core co-expressed biosynthetic enzymes related to the red stem phenotype. Real-time quantitative PCR (qRT-PCR) analysis confirmed that these six candidate genes displayed tissue-specific expression patterns consistent with the transcriptomic data (Fig. 3E), supporting their potential roles in anthocyanin biosynthesis associated with the red stem phenotype in AMM.

### Functional characterization of AmC4H, AmCHS, and AmCHI

**Figure 4:**
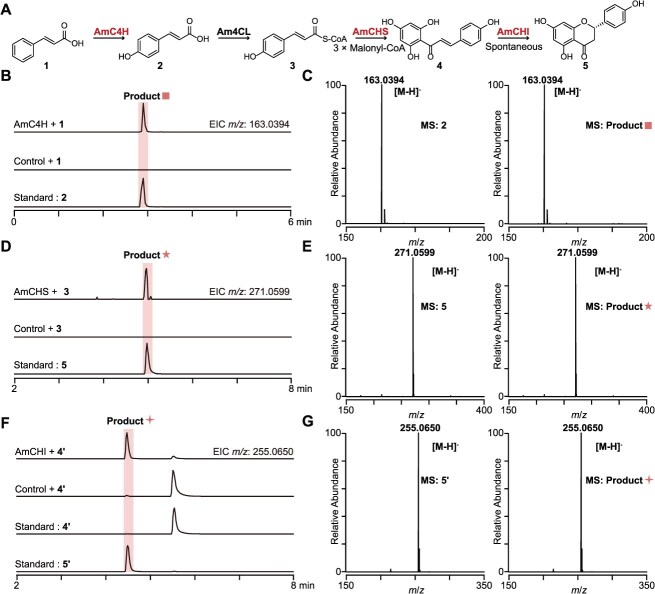
Functional characterization of AmC4H, AmCHS, and AmCHI. (A) Catalytic steps of the three enzymes. (B, C) AmC4H catalytic conversion of **1** to **2** and the products were analyzed by HPLC and MS. (D, E) AmCHS catalytic conversion of **3** to **4 (5)** and the products were analyzed by HPLC and MS. (F, G) AmCHI catalytic conversion of **4**′ to **5**′ products were analyzed by HPLC and MS.

To validate the enzymatic functions of candidate genes involved in the early steps of anthocyanin biosynthesis, we performed heterologous expression and *in vitro* enzyme assays for *AmC4H*, *AmCHS*, and *AmCHI* (Fig. 4A). The coding sequence of *AmC4H* was cloned into the pESC-His vector and expressed in *Saccharomyces cerevisiae* WAT11 cells. Meanwhile, *AmCHS* and *AmCHI* were cloned into the pET28a vector and expressed in *Escherichia coli* BL21 (DE3). Microsomal fractions were isolated from WAT11 cells harboring AmC4H, and enzyme activity assays confirmed that AmC4H catalyzed the conversion of cinnamic acid (**1**) to *p*-coumaric acid (**2**) (Fig. 4B and C).

For AmCHS and AmCHI, the recombinant proteins were purified using Ni-NTA affinity chromatography. *In vitro* assays showed that AmCHS catalyzed the synthesis of naringenin (**5**) from *p*-coumaroyl-CoA (**3**) (Fig. 4D and E). As the intermediate product naringenin chalcone (**4**) rapidly undergoes spontaneous isomerization in the reaction system, only **5** was detected as the final product. To evaluate AmCHI activity, isoliquiritigenin (**4′**) was used as an alternative substrate to avoid detection issues with **4**, a reaction that also represents a step in isoflavonoid biosynthesis. The reaction product, liquiritigenin (**5′**), was successfully detected, suggesting that AmCHI possesses chalcone isomerase activity (Fig. 4F and G).

### Functional characterization of hydroxylase genes *AmF3*′*H* and *AmF3H*

**Figure 5:**
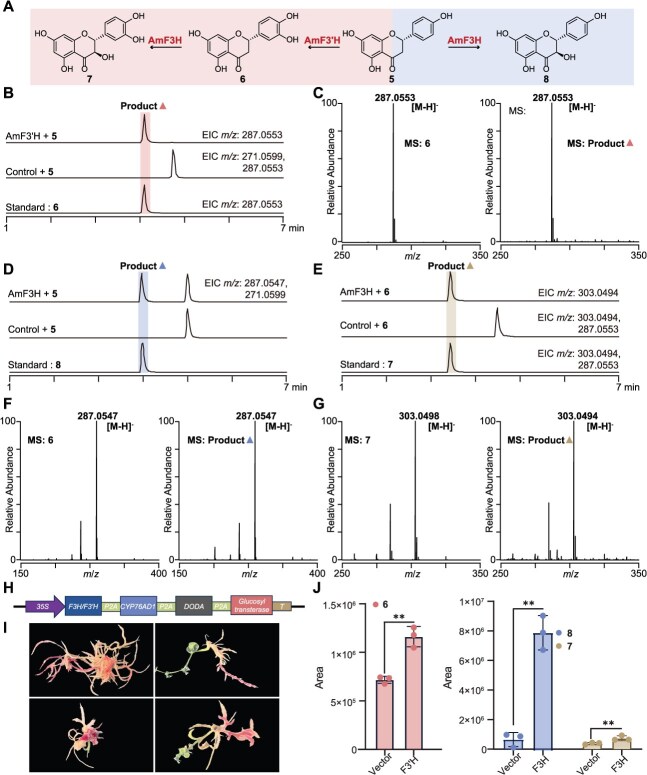
Functional characterization of AmF3′H and AmF3H. (A) Catalytic steps of AmF3′H and AmF3H. (B, C) AmF3′H catalytic conversion of **5** to **6** products were analyzed by HPLC and MS. (D, E) AmF3H catalytic conversion of **5** to **8** and **6** to **7** were analyzed by HPLC. (F, G) AmF3H catalytic products analyzed by MS. (H) Vector constructs used for transgenic hairy root expression. (I) A picture of transgenic hairy roots of AMM. (J) Metabolite levels in hairy roots overexpressing AmF3′H or AmF3H and in empty vector controls, detected by LC/MS.

To investigate the enzymatic roles of *AmF3*′*H* and *AmF3H*, two key hydroxylases involved in the midstream steps of anthocyanin biosynthesis, we performed both *in vitro* and *in vivo* functional assays (Fig. 5A). *AmF3*′*H* was heterologously expressed in the yeast strain WAT11 to obtain microsomal fractions, while *AmF3H* was expressed in *E*. *coli* BL21(DE3) and purified via affinity chromatography. *In vitro* enzyme assays revealed that AmF3′H catalyzed the conversion of **5** to eriodictyol (**6**) (Fig. 5B and C), whereas AmF3H catalyzed the hydroxylation of **5** and **6** to produce dihydrokaempferol (**8**) and dihydroquercetin (**7**), respectively (Fig. 5D-G).

To further validate their functions in planta, we constructed expression vectors in which each gene was fused to the RUBY reporter system via a P2A linker under the control of the 35S promoter (Fig. 5H). The RUBY system enables *in vivo* visualization by coupling the key enzymes CYP76AD1, DODA, and glycosyltransferases to produce red betalain pigments without the need for exogenous substrates. The constructs were introduced into hypocotyl explants of AMM seedlings using the *Agrobacterium rhizogenes* strain ArA4, which resulted in the generation of transgenic hairy roots (Fig. 5I). LC/MS analysis showed that, compared to the empty vector control, AmF3′H overexpression led to a marked increase in eriodictyol levels, while AmF3H overexpression resulted in 8.19-fold and 1.89-fold increases in apigenin and dihydroquercetin levels, respectively (Fig. 5J). Collectively, these *in vitro* and *in vivo* results demonstrate that AmF3′H and AmF3H function as key catalytic enzymes in the anthocyanin biosynthetic pathway in AMM.

### Catalytic properties and structural basis of AmBZ1

**Figure 6:**
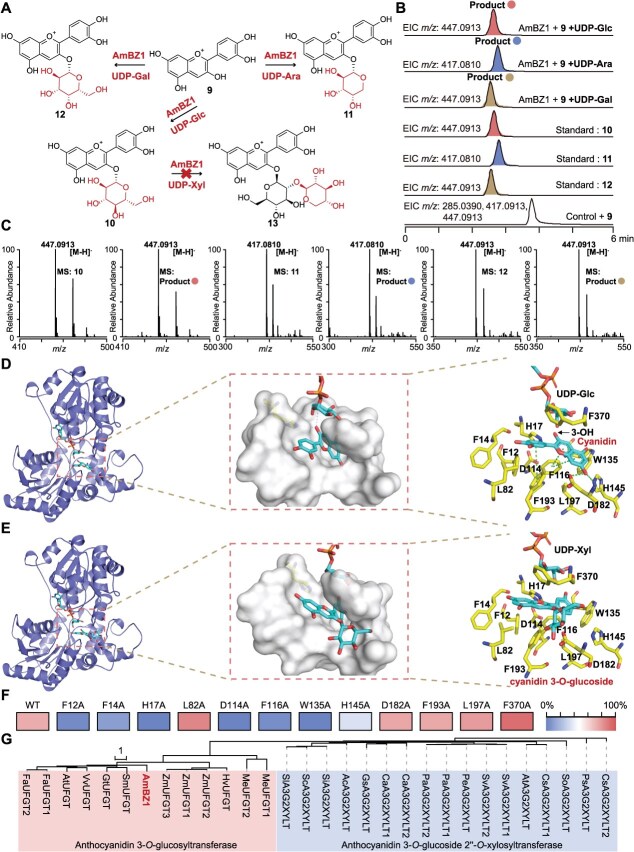
Functional characterization of AmBZ1. (A) Proposed catalytic role of AmBZ1 in cyanidin glycosylation. (B, C) HPLC and MS analysis of the glycosylated products generated by AmBZ1. (D) Molecular docking of cyanidin with AmBZ1. (E) Molecular docking of cyanidin-3-*O*-glucoside with AmBZ1. (F) Relative conversion rates of 12 alanine-substituted AmBZ1 mutants for the biosynthesis of cyanidin-3-*O*-arabinoside. (G) Phylogenetic analysis of functionally characterized anthocyanidin 3-*O*-glucosyltransferases and anthocyanidin 3-*O*-glucoside 2″-*O*-xylosyltransferases. Reference protein sequences used in this analysis are listed in Table S11.

Glycosylation, as the terminal modification step in anthocyanin biosynthesis, plays a critical role in stabilizing anthocyanins and enhancing their solubility. To investigate the function of *AmBZ1*, we cloned the gene into the pET28a vector and expressed it in *E. coli* BL21(DE3) to obtain recombinant protein. Based on the metabolomic results that highlighted cyanidin as a core metabolite associated with the red stem phenotype, *in vitro* enzymatic assays were performed using cyanidin as the substrate and UDP-Glc, UDP-Ara, and UDP-Gal as sugar donors (Fig. 6A). The results demonstrated that AmBZ1 catalyzed the formation of three distinct glycosylated products, indicating broad glycosyltransferase activity toward cyanidin (Fig. 6B and C).

Metabolomic analysis revealed that the content of cyanidin 3-sambubioside was significantly higher in RS than in GS. Its biosynthesis involves xylosylation at the 2-position of the glucose moiety in cyanidin 3-*O*-glucoside. To evaluate whether AmBZ1 could catalyze this step, *in vitro* assays were conducted using cyanidin 3-*O*-glucoside as the substrate and UDP-Xyl as the sugar donor (Fig. S10). However, AmBZ1 failed to glycosylate cyanidin 3-*O*-glucoside. Thus, the observed difference in cyanidin 3-sambubioside levels between RS and GS may be attributed to variations in the accumulation of its precursor, cyanidin 3-*O*-glucoside.

To elucidate structural basis of the substrate selectivity of AmBZ1, a three-dimensional homology model was constructed using Alphafold 3 [29]. Molecular docking analyses were performed with cyanidin and cyanidin-3-*O*-glucoside. The results indicated that cyanidin can stably bind to the active pocket of AmBZ1, with its A ring and C ring points to the interior and entrance of the substrate binding pocket, respectively (Fig. 6D). A series of interactions were observed between cyanidin and surrounded residues, including hydrogen bonds formed by the 7-OH group with H145 and D182, as well as π–π stacking interactions between F193 and the B ring, and between F116 and the A/C rings. In the modeled structure, the 3-OH of cyanidin is positioned close to the key catalytic residue H17 and is oriented toward the anomeric carbon of the UDP-Glc. The glycosylation reaction catalyzed by AmBZ1 is initiated by the H17-mediated deprotonation of the 3-OH group, followed by an S_N_2 nucleophilic substitution between the aglycone and UDP-Glc to generate the glycosylated product.

However, due to the narrow substrate binding pocket, cyanidin-3-*O*-glucoside is unable to form a suitable binding conformation with AmBZ1 for the glycosylation. In contrast to cyanidin, the A and C rings of cyanidin-3-*O*-glucoside are oriented toward the entrance and interior of the substrate binding pocket, respectively (Fig. 6E). The 3-glucosyl moiety is positioned outside the catalytic center, and no hydroxyl group is placed in proximity to the catalytic residue H17 because of the change of the binding conformation. Therefore, AmBZ1 could not accommodate cyanidin-3-*O*-glucoside as the substrate for further glycosylation.

To further validate the structural basis of this substrate specificity, we conducted alanine substitution mutagenesis on key residues within the active pocket of AmBZ1. Mutations at H17, D114, and W135 completely abolished its catalytic activity toward converting cyanidin into cyanidin-3-*O*-arabinoside. In addition, several other mutants exhibited reduced catalytic activities, indicating that these surrounding residues play critical roles in substrate binding recognition (Fig. 6F). Furthermore, none of the mutants acquired the ability to catalyze the conversion of cyanidin-3-O-glucoside into cyanidin-3-sambubioside (Fig. S11), suggesting that altering individual residues is insufficient to reprogram the enzyme′s substrate preference. This indicates that the exclusion of glycosylated anthocyanins may be governed by a more global structural constraint rather than by a few catalytic residues alone.

In line with this, a phylogenetic analysis of functionally characterized glycosyltransferases revealed that anthocyanidin 3-*O*-glucosyltransferases and anthocyanidin 3-*O*-glucoside 2″-*O*-xylosyltransferases form two distinct clades (Fig. 6G). AmBZ1 clusters with the former group, which typically utilize aglycone anthocyanidins as acceptors rather than glucosylated intermediates. This evolutionary divergence further explains why the catalytic function of AmBZ1 cannot be easily modified to accommodate additional sugar donors through site-directed mutagenesis.

Taken together, these findings demonstrate that AmBZ1 plays a crucial catalytic role in the biosynthesis of cyanidin-based anthocyanins.

## Discussion

### Association between stem color variation and root metabolite profiles in AMM

Plant phenotypes are the external manifestations of interactions between genotype and environmental factors, and their variations are often accompanied by systematic changes in the composition and abundance of secondary metabolites. Studies have shown that alterations in aboveground phenotypic traits can influence the metabolic profile of underground organs through signal transduction, metabolite transport, or resource reallocation pathways [30]. For instance, in *Populus tremuloides*, leaf wounding leads to significant changes in the composition and levels of salicylic acid derivatives in the roots, suggesting the presence of a highly coordinated systemic response network across plant organs [31]. In the present study, stem color variation in AMM did not only reflect differences in external morphology but also correspond with distinct accumulation patterns of key active compounds in the roots. The contents of isoflavones, such as calycosin 7-*O*-glucoside, were consistently higher in RR than in GR, and the overall abundance of volatile metabolites was also elevated in RR. These findings suggest a cross-organ association between stem coloration and the accumulation of root secondary metabolites. Altogether, stem color shows potential as a visible phenotypic reference associated with the accumulation of medicinally important compounds in AMM, and provides a practical basis for the precise selection and standardized breeding of AMM germplasm.

Similar phenotype-component associations have been reported in other medicinal plants. For example, in *Salvia miltiorrhiza*, the red coloration of roots is closely associated with the accumulation of tanshinones [32]. In *Atractylodes lancea*, the accumulation of polyacetylenes such as atractylodin and (4E,6E,12E)-tetradecatriene-8,10-diyne-1,3-diol-diacetate is significantly correlated with red pigmentation in the rhizome oil chambers, further supporting a tight linkage between visible traits and bioactive metabolite profiles [33]. In comparison, this study in AMM integrates transcriptomic and metabolomic analyses with functional validation of key biosynthetic genes, uncovering a potential co-remodeling mechanism between the anthocyanin and isoflavone branches of the flavonoid pathway. This integrative approach provides a biosynthetic explanation for the observed association between stem color and root compound levels, and offers a conceptual framework for dissecting the coordinated regulation between visible traits and medicinal quality in herbal plants.

### Red stem formation is linked to cyanidin-type anthocyanin accumulation and tissue-specific expression of biosynthetic genes

The coloration of plant tissues is primarily determined by the composition and accumulation levels of pigments such as anthocyanins [34]. In this study, cyanidin derivatives including cyanidin 3-*O*-glucoside were specifically enriched in RS, indicating that the cyanidin biosynthetic branch is activated in RS, which in turn contributes to the red stem phenotype. This observation is consistent with previous findings in *Camellia oleifera* flowers [35] and the seasonal color change in *Acer palmatum* leaves [36], both supporting the central role of cyanidin in organ pigmentation.

The tissue-specific distribution of anthocyanins in plants is typically associated with the spatial expression patterns of genes in the biosynthetic pathway [37]. In our study, several key genes involved in cyanidin biosynthesis, including *AmC4H*, *AmCHS*, *AmCHI*, *AmF3H*, *AmF3′H*, and *AmBZ1*, were significantly upregulated in RS. Both *in vitro* enzymatic assays and hairy root transformation confirmed the catalytic activity of these gene products, demonstrating their ability to drive the accumulation of cyanidin intermediates and end products. Among them, *AmCHS* and *AmCHI* are essential nodes in the flavonoid biosynthesis pathway, and their expression dynamics can substantially influence the overall flavonoid metabolic profile [38]. Moreover, the expression trends of cyanidin biosynthetic genes were consistent with the accumulation patterns of cyanidin-related metabolites, suggesting the pathway is transcriptionally regulated *in vivo*. Previous studies have shown that R2R3-MYB, bHLH, and WD40 proteins can form the MBW transcriptional complex, which binds to cis-elements in the promoters of target genes to regulate their spatial and temporal expression [39]. This regulatory mechanism has been well documented in various plant species. For instance, in maize, the R2R3-MYB transcription factor *p1* specifically regulates the expression of anthocyanin biosynthetic genes in the pericarp and floral organs, thus determining the spatial distribution and localized accumulation of pigments [40]. Together, our findings suggest that the red stem phenotype in AMM results from high-level expression of cyanidin biosynthetic genes in stem tissues and the consequent tissue-specific accumulation of cyanidin-type anthocyanins. Nevertheless, this phenotype may also be influenced by a complex network involving transcriptional regulation, hormonal signaling, and epigenetic modifications, which remains to be fully elucidated.

### Coordinated remodeling of the flavonoid metabolic pathway links red stem formation with the accumulation of bioactive compounds in roots

**Figure 7:**
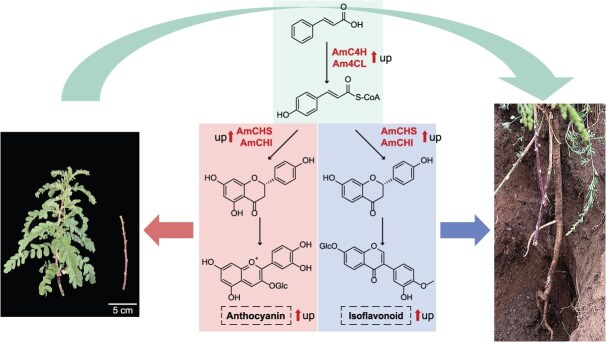
Schematic model illustrating the metabolic and transcriptional coordination underlying stem coloration and root isoflavone accumulation in AMM.

In addition to the accumulation of anthocyanin compounds, this study revealed a broader metabolic reprogramming in red-stemmed AMM. Both metabolomics and transcriptomic analyses demonstrated that, compared with GS, RS exhibited significantly elevated levels of multiple flavonoid- and phenylpropanoid-related compounds and genes in both roots and stems. These findings suggest that stem color variation is associated with a global adjustment of the plant's secondary metabolic network, rather than a localized change in the anthocyanin branch alone. Previous studies have shown that the flavonoid metabolic network can undergo large-scale reprogramming via upstream carbon flux redistribution and coordinated regulation of multiple pathway branches, involving transcriptional, post-transcriptional, and epigenetic mechanisms [41]. Notably, both anthocyanins and isoflavones are subclasses of flavonoids and share several core enzymes in the upstream biosynthetic pathway, such as CHS and CHI. In RS, the significant upregulation of these shared genes may not only enhance metabolic flow toward cyanidin accumulation but also increase precursor availability and overall throughput of the flavonoid network. This coordinated gene expression likely promotes the biosynthesis and accumulation of various downstream metabolites, including root-specific isoflavones (Fig. 7). Moreover, phenylpropanoid precursors have been shown to be mobile between different plant tissues [42], suggesting that changes in stem-localized metabolism may influence the broader flavonoid metabolic landscape, including in root-specific branches, through inter-organ precursor transport. However, to date, no specific transporters have been functionally characterized in AMM to mediate such long-distance phenylpropanoid translocation between stems and roots. Therefore, while our results support a strong correlation between stem pigmentation and root chemistry, the underlying mechanisms remain hypothetical.

### Potential applications of stem color as a visible phenotypic reference.

Through integrated multi-omics analyses, this study reveals a coupling relationship between stem color and the accumulation of medicinal compounds in the roots of AMM, suggesting that stem color may serve as a visible phenotypic proxy for predicting variation in root phytochemical profiles. Similar correlations between visible phenotypes and metabolite profiles have been reported in *Perilla frutescens*, where purple morphotypes typically accumulate more flavonoids and essential oils than green ones [43, 44]. Given its high visibility and ease of recognition, stem color offers practical advantages for high-throughput screening in the field and holds application potential in germplasm evaluation, cultivar traceability, and classification. Moreover, the anthocyanin enrichment observed in RS may not only confer distinct coloration but also enhance plant resistance to UV radiation and pathogens, thereby strengthening defense responses [45]. In addition, anthocyanins possess diverse biological activities, such as antioxidant and anti-inflammatory effects [46]. The aerial parts of red-stemmed AMM, rich in anthocyanins, could be utilized as feed additives to improve animal health and increase the nutritional quality and storage stability of forage. Collectively, these findings underscore the significance of stem color as a valuable phenotypic trait for the development and utilization of AMM germplasm resources. Nevertheless, we acknowledge that the correlation between stem coloration and root phytochemical traits may be influenced by genetic background and environmental factors, and further population-based validation will be necessary before large-scale application.

## Conclusion

This study systematically compared the root compositions of AMM with different stem colors and found that RR exhibited significantly higher levels of isoflavones and volatile compounds. Metabolomic and transcriptomic analyses revealed that the substantial accumulation of cyanidin-based anthocyanins in red-stem tissues serves as the metabolic basis for the formation of this phenotype. This accumulation is closely associated with the upregulated expression of key genes in the anthocyanin biosynthetic pathway, including *AmF3′H*, *AmF3H*, and *AmBZ1*. These findings provide a theoretical foundation for considering stem color as a visible phenotypic reference associated with phytochemical variation in AMM, and offer molecular insights into the relationship between metabolite accumulation and phenotypic differentiation.

## Materials and methods

### Plant materials and culture conditions

Six RS and six GS AMM plants with uniform growth were collected from a plantation in Hunyuan County. The roots of three plants from each group were used for VOCs analysis, and the remaining three were air-dried, ground, and sieved for the determination of key quality control indicators specified in the Chinese pharmacopeia for Huangqi. AMM seeds were grown individually in pots placed in a growth chamber set at 23°C, 70% humidity, with a light/dark cycle of 14/10 h. Three uniformly growing plants of each stem color were selected, and their stem bark and roots were immediately frozen in liquid nitrogen and stored at −80°C for transcriptomic and metabolomic analysis.

### Quality control index detection

The content of four bioactive isoflavones was determined using HPLC-UV with a mobile phase of acetonitrile (A) and 0.2% formic acid solution (B), gradient elution (0–25 min, 15%–32% A; 25–38 min, 32%–47.6% A; 38–38.5 min, 47.6%–90% A; 38.5–43 min, 90% A; 43–43.1 min, 90%–15% A; 43.1–44 min, 15% A); column temperature at 40°C, flow rate of 1.0 ml/min, injection volume of 10 μl, and detection wavelength of 254 nm.

### Detection of VOCs

GC-IMS (FlavourSpec® 25; Gesellschaft für analytische Sensorsysteme mbH, Dortmund, Germany) was used for the detection of VOCs with an analysis time of 20 min; the column was MXT-5 (15 m × 0.53 mm × 1.0 μm); column temperature was 60°C; carrier gas/drift gas was N_2_; IMS drift tube temperature was 45°C; drift gas control mode was constant flow at 150 ml/min; carrier gas flow rate started at 2 ml/min for 2 min, then linearly increased to 100 ml/min over 2 to 10 min, and maintained at 150 ml/min for 10 to 20 min.

### Metabolite extraction and metabolomic analysis

An aliquot of 50 mg of freeze-dried plant sample was mixed with 1000 μl extraction solvent (methanol: acetonitrile: water = 1:2:1) and vortexed for 30 seconds. Steel balls were added, followed by grinding at 45 Hz for 10 min and ultrasonic treatment for 10 min (ice-water bath). Samples were left to stand at −20°C for 1 h, then centrifuged at 4°C, 12 000 rpm for 15 min. Then, 300 μl of the supernatant was filtered through a 0.22-μm organic filter membrane into a 2-ml sample bottle for metabolomic analysis.

UPLC-ESI-MS/MS System for Metabolomic Analysis (UPLC: Waters Acquity I-Class Plus; MS: Applied Biosystems QTRAP 6500+): UPLC conditions: Waters HSS-T3 column (1.8 μm, 2.1 mm × 100 mm); mobile phase A (0.1% formic acid and 5 mM ammonium acetate in water) and mobile phase B (0.1% formic acid in acetonitrile). Gradient elution: 0–1.5 min, 98% A; 1.5–5 min, 98%–50% A; 5–9 min, 50%–2% A; 9–10 min, 2% A; 10–11 min, 2%–98% A; 11–14 min, 98% A. Flow rate, 0.35 ml/min; column temperature, 50°C; injection volume, 4 μl. MS parameters: ESI source temperature 550°C, ion spray voltage (IS) 5500 V (positive mode)/−4500 V (negative mode), ion source gas I (GSI), II (GSII), and curtain gas (CUR) set at 50, 55, and 35 psi, respectively. Collision-induced dissociation parameters set to medium. Instrument tuning and mass calibration with 10 and 100 μmol/l polypropylene glycol solutions in QQQ and LIT modes, respectively. QQQ scans used MRM mode with nitrogen as the collision gas set to medium. DP and CE for each MRM ion pair optimized, monitoring specific MRM ion pairs for each elution period.

Metabolite identification was performed in the MRM mode by UPLC-ESI-MS/MS, based on a self-established software database (GB-PLANT). PCA and Spearman correlation analysis were used to assess sample reproducibility and quantitative control samples. Identified compounds were classified and pathway information retrieved from KEGG, HMDB, and LIPID MAPS databases. Fold change was calculated based on grouping information, and *t*-tests were used to compute the significance *P* value for each compound. OPLS-DA modeling was conducted using the R package ropls, with 200 permutation tests validating the model's reliability and VIP values calculated through multiple cross-validation. Metabolites with FC > 2 and VIP > 1 were considered DAMs.

### Transcriptome analysis

Total RNA was extracted using the RNA prep Pure Plant Kit (Tiangen, Beijing, China). RNA concentration and purity were measured with a NanoDrop 2000 (Thermo Fisher Scientific, Wilmington, DE), and RNA integrity was assessed with the RNA Nano 6000 Assay Kit on an Agilent Bioanalyzer 2100 system (Agilent Technologies, CA, USA). Sequencing libraries were prepared using the Hieff NGS Ultima Dual-mode mRNA Library Prep Kit for Illumina (Yeasen Biotechnology, Shanghai, China) according to the manufacturer's instructions, with indexes added to each sample’s sequence. Libraries were sequenced on an Illumina NovaSeq platform, generating 150 bp paired-end reads. Clean reads obtained after quality control were aligned to the AMM reference genome [47] using Hisat2 software. Known transcripts were identified and new transcripts predicted with StringTie software. Gene functions were annotated based on the following databases: Nr (NCBI non-redundant protein sequences), Pfam (Protein family), KOG/COG (Clusters of Orthologous Groups of proteins), Swiss-Prot (A manually annotated and reviewed protein sequence database), KO (KEGG Ortholog database), and GO (Gene Ontology). Gene expression levels were estimated as fragments per kilobase of transcript per million fragments mapped (FPKM). Differential expression analysis between the two groups was performed using DESeq2, with adjusted *P* values obtained through the Benjamini and Hochberg method for controlling the false discovery rate. Genes with false discovery rate (FDR) <0.05 and FC ≥2 were designated as differentially expressed. GO and KEGG enrichment analyses of DEGs were performed using clusterProfiler and KOBAS.

### Real-time quantitative PCR

qRT-PCR analysis was performed on the stem and root tissues of different stem-colored AMM using primers listed in Table S12.

### WGCNA analysis

WGCNA was performed using the R-package WGCNA. Initially, DEGs between the GS and RS groups were selected, with low-variance genes removed. The R-package WGCNA was then used to construct a weighted gene co-expression network, with a soft thresholding power (β = 6) chosen to ensure a scale-free topology. Hierarchical clustering was applied to partition the genes into distinct modules, and the correlation between each module and the phenotype was calculated. Finally, hub genes within each module were identified based on module membership (kME ≥ 0.8).

### Cloning of candidate genes

The CDSs of candidate genes were amplified from cDNA using TransStart FastPfu DNA Polymerase (TransGen, China). The CDSs of *AmCHS*, *AmCHI*, *AmF3H*, and *AmBZ1* were cloned into the pET-28a(+) vector (Invitrogen, USA) at the BamHI and HindIII restriction sites. The CDSs of *AmC4H* and *AmF3*′*H* were inserted into the pESC-His vector (Invitrogen, USA) at the BamHI site. The primer sequences used in this study are listed in Table S13.

### Expression of candidate genes

The verified recombinant plasmids containing AmCHS, AmCHI, AmF3H, and AmBZ1 were transformed into *Escherichia coli* BL21(DE3) competent cells (TransGen Biotech, China) for protein expression. Single colonies were inoculated into LB liquid medium supplemented with 50 μg/ml kanamycin and cultured at 37°C with shaking at 180 rpm until the optical density at 600 nm (OD_600_) reached 0.6 to 0.8. Protein expression was induced by adding 0.1 mM isopropyl-β-d-thiogalactopyranoside (IPTG) and incubating the cultures at 18°C for 20 h. Bacterial cells were harvested by centrifugation and resuspended in 20 ml lysis buffer (50 mM NaH₂PO₄, 300 mM NaCl, 10 mM imidazole, pH 8.0). Cell lysis was performed by sonication on ice, and the supernatant was collected for nickel affinity chromatography. Gradient elution was carried out using elution buffers containing 30 mM and 250 mM imidazole (in 50 mM NaH₂PO₄, 300 mM NaCl, pH 8.0). The eluted fractions were collected, mixed with 25% (v/v) glycerol, and stored at −80°C for further use.

The recombinant plasmids harboring AmC4H and AmF3′H were introduced into the yeast strain *Saccharomyces cerevisiae* WAT11 for heterologous expression. Transformed yeast cells were cultured in synthetic dropout (SD) medium lacking histidine (SD-His). Liquid cultures were initiated by inoculating a single colony into 50 ml of SD-His medium containing 20 g/l glucose and incubated overnight at 28°C. Cells were then harvested by centrifugation (1000 × g, 2 min) and resuspended in 25 ml of SD-His supplemented with 20 g/l galactose to induce protein expression. Induction was carried out at 28°C for 24 to 48 h. Yeast microsomes were prepared as previously described.

### Molecular docking

The structure model of AmBZ1 was constructed by Alphafold 3. Molecule docking of AmBZ1 with substrates were carried out by Autodock [48]. A total of 100 docking conformations were generated for each substrate. The docking conformations of the lowest docking scores were used for further analysis.

### Site-directed mutagenesis

Site-directed mutagenesis was performed on pET28a-AmBZ1 constructs using PCR-based methods to introduce point mutations at designated residues. All mutations were verified by Sanger sequencing. Expression and purification of mutant proteins were conducted using the same protocols as for wild-type constructs.

## Supplementary Material

Web_Material_uhag088

## Data Availability

The raw transcriptome sequencing data have been deposited into the China National Center for Bioinformation (CNCB) at the following URL: http://www.gpgenome.com/species/109.
