# Supplementary material for: Elucidating the mechanisms underlying differential anthocyanin biosynthesis and its link to stem color and root isoflavonoid levels in Astragalus membranaceus var. mongholicus
Source: Hortic Res. 2026 Mar 4;13(6):uhag088. doi: 10.1093/hr/uhag088 (PMC13254205; doi:10.1093/hr/uhag088)
Supplement: Web_Material_uhag088 [file web_material_uhag088.zip › Supplemental information-HR-R1.pdf]

Supplementary Information

Elucidating the Mechanisms Underlying Differential Anthocyanin Biosynthesis and Its Link to Stem Color and Root Isoflavone Levels in *Astragalus membranaceus* var. *mongholicus*

Chen Yi[1], Sifei Duan[2], Meng Zhang[1], Yang-oujie Bao[1], Yungang Tian[1], Xuehui Dong[2], Min Ye[1*]

[1] State Key Laboratory of Natural and Biomimetic Drugs, School of Pharmaceutical Sciences, Peking University, Beijing 100191, China

[2] College of Agronomy and Biotechnology, China Agricultural University, Beijing 100193, China

*Correspondence: Min Ye (yemin@bjmu.edu.cn)

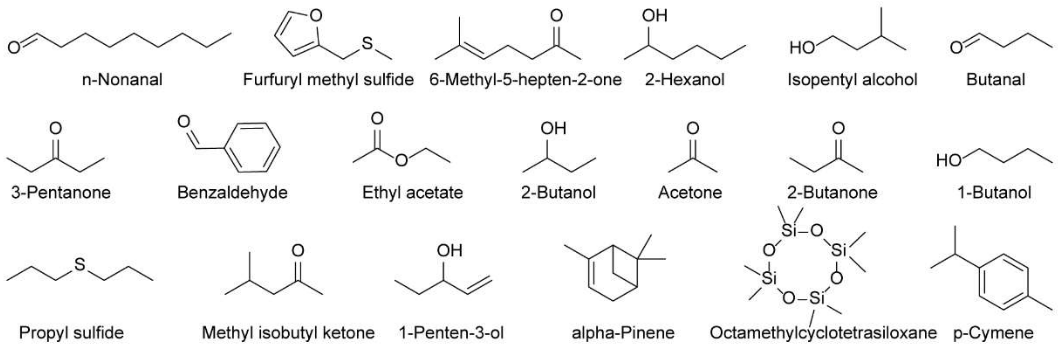

Supplementary Figure 1. Structures of the volatile organic compounds (VOCs).

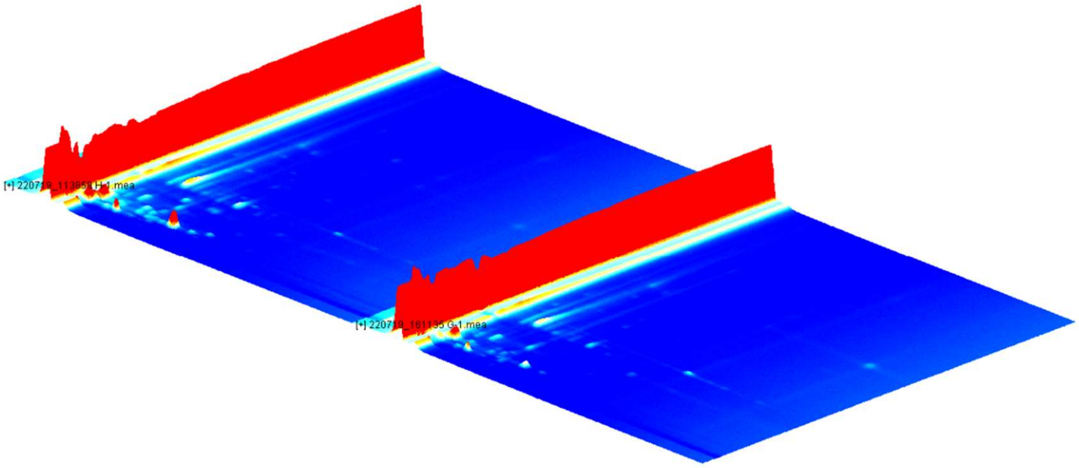

Supplementary Figure 2. 3D GC-IMS spectra of VOCs in the roots of AMM with different stem colors.

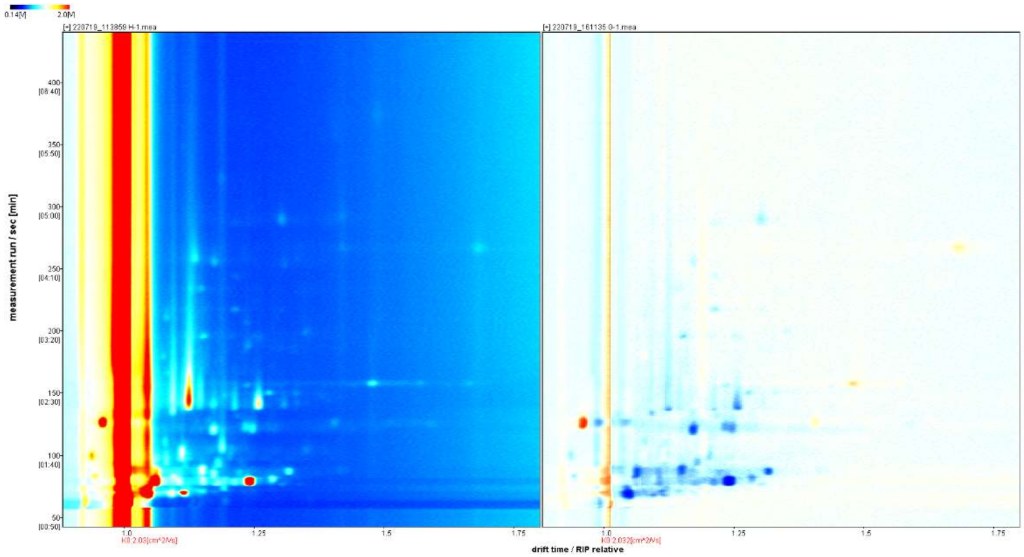

Supplementary Figure 3. Comparative GC-IMS spectra of VOCs in the roots of AMM with different stem colors.

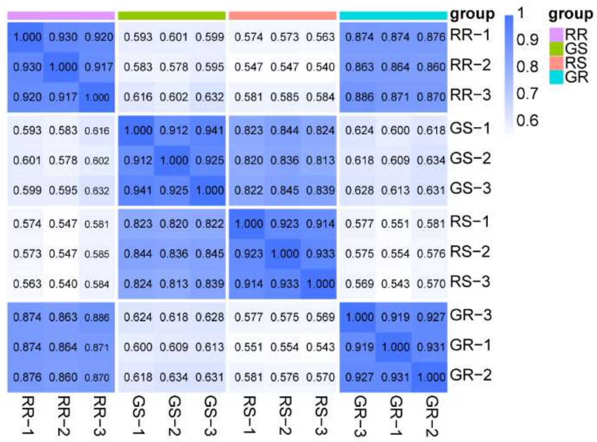

Supplementary Figure 4. Correlation analysis of different samples.

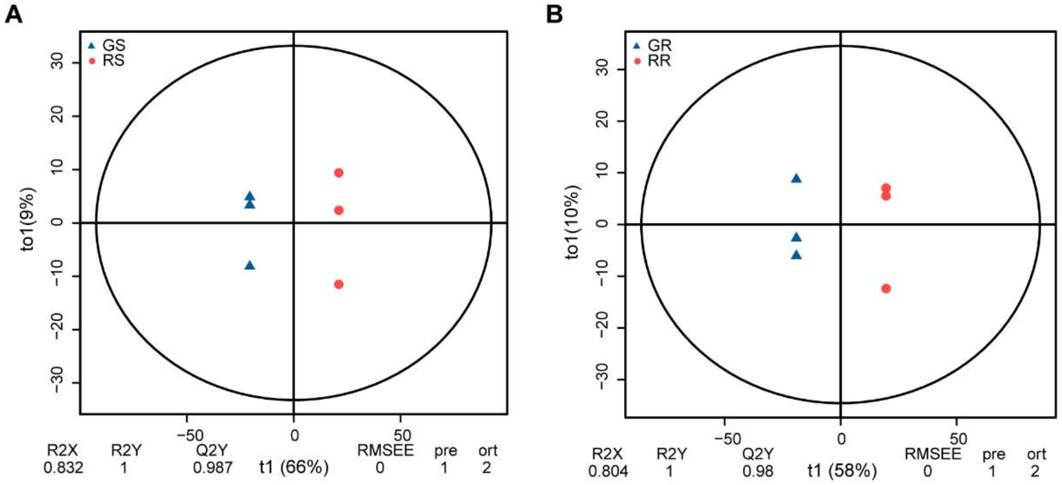

Supplementary Figure 5. OPLS-DA analysis of GS *vs*. RS (A) and GR *vs*. RR (B) comparison groups.

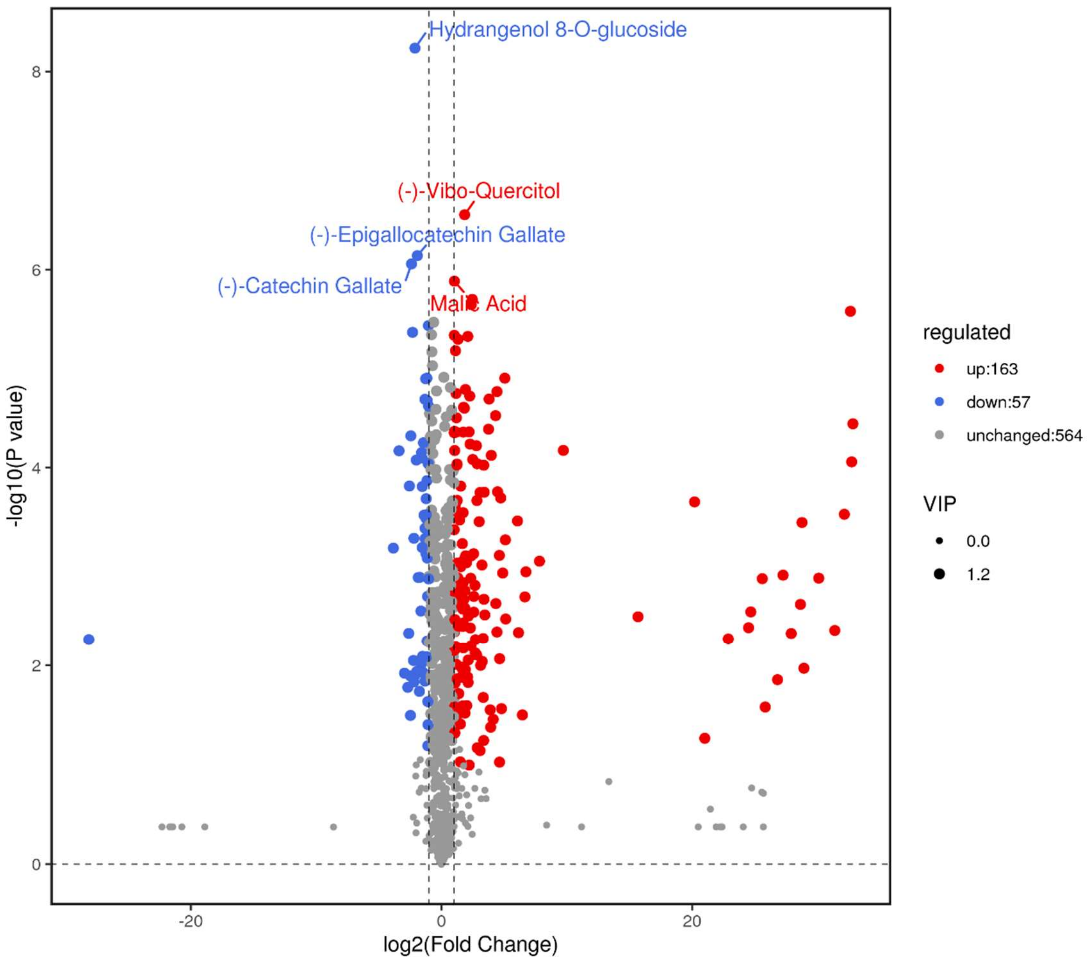

Supplementary Figure 6. Volcano plot of differential metabolites between GS and RS groups.

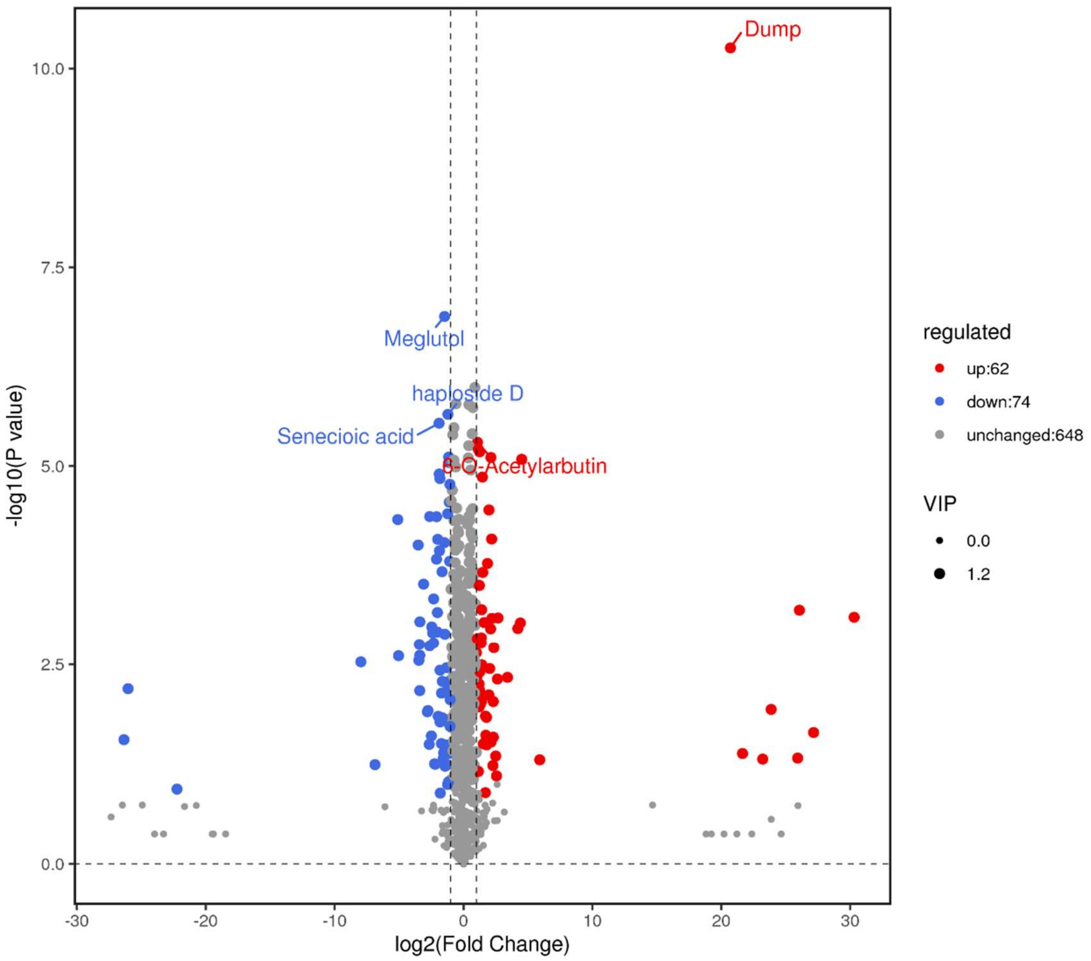

Supplementary Figure 7. Volcano plot of differential metabolites between GR and RR groups.

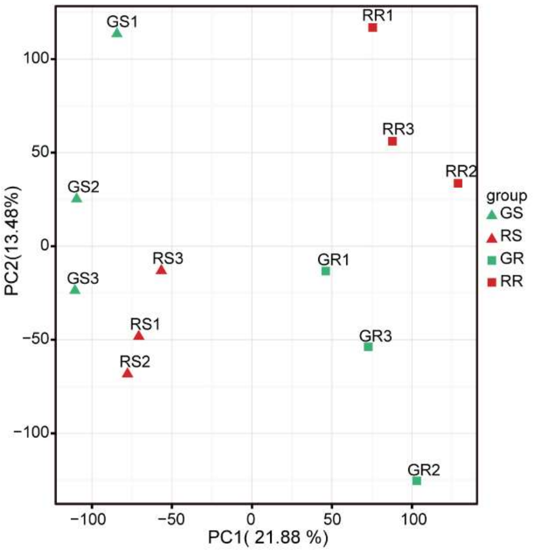

Supplementary Figure 8. PCA analysis of different samples.

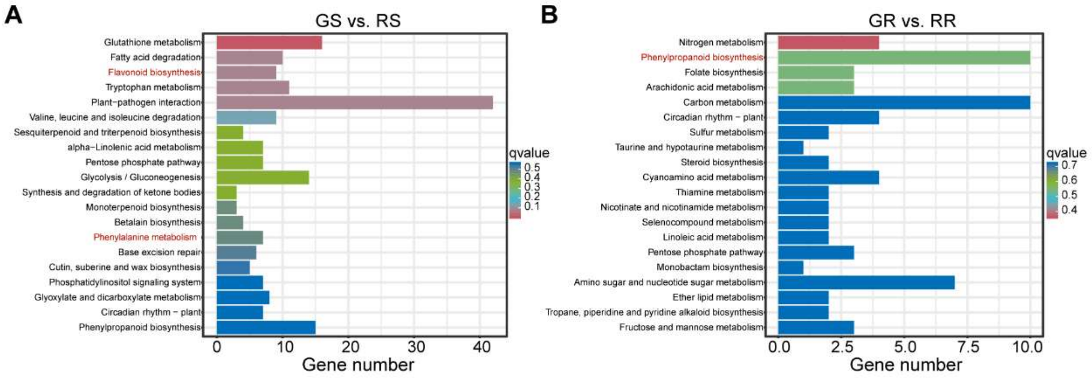

Supplementary Figure 9. KEGG enrichment analysis of differentially expressed genes in GS *vs*. RS (A) and GR *vs*. RR (B) comparison group.

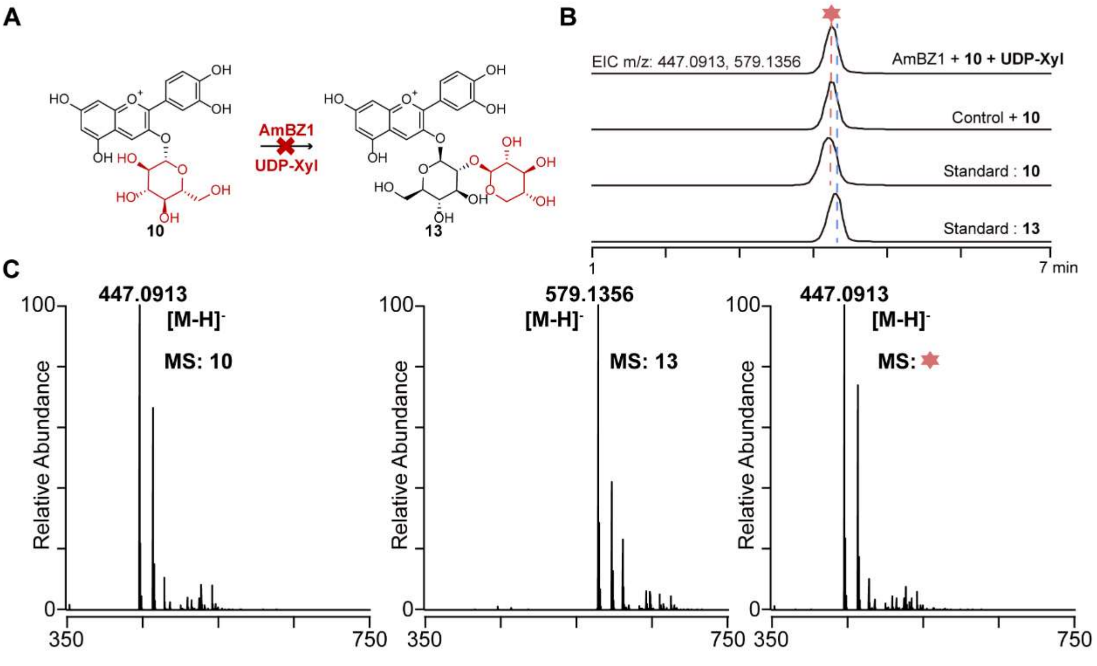

Supplementary Figure 10. Catalytic inactivity of AmBZ1 evidenced by co-elution and MS. (A) Reaction scheme. (B) HPLC with unresolved peaks. (C) Key MS comparison excluding product formation.

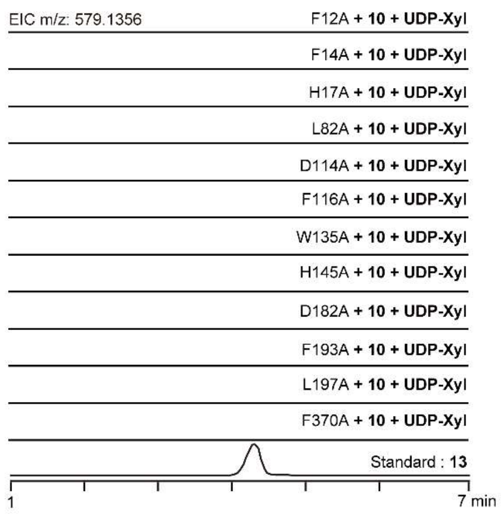

Supplementary Figure 11. In vitro catalytic activity of 12 alanine-substituted AmBZ1 mutants using cyanidin-3-*O*-glucoside (**10**) as substrate.

Supplementary Table 1. Overview of natural AMM populations and germplasm accessions.

Serial number	Population	Number of germplasm accessions	Province
1	BHY	19	Shanxi
2	LKS	12	Shanxi
3	YEQ	23	Shanxi
4	MSLC	38	Shanxi
5	MSJQ	22	Shanxi
6	PQG	28	Shanxi
7	DSG	14	Shanxi
8	LYS-gjg	25	Shanxi
9	LYS-dp	22	Shanxi
10	LQG	8	Shanxi
11	XSZ	22	Shanxi
12	HYS	16	Shanxi
13	ZJY	19	Shanxi
14	DLG	12	Shanxi
15	DY-yjc	30	Shanxi
16	NTC	4	Shanxi
17	HCS	3	Shanxi
18	GZ-tyg	9	Shanxi
19	SHC-dd	35	Heibei
20	SHG	10	Shaanxi
21	CG	22	Shaanxi
22	XBJ	7	Gansu
23	XNGMJ	14	Gansu
24	XNG	4	Gansu
25	KLCMJ	32	Gansu
26	KLC	9	Gansu
27	MEG	21	Gansu
28	YW	42	Gansu
29	SJG	3	Gansu
30	FSL	43	Gansu
31	TBS	5	Gansu
32	LSLC	33	Heilongjiang
33	SBZLC	36	Heilongjiang
34	XJJ	2	Heilongjiang
35	EHFZ	21	Heilongjiang
36	HH	5	Heilongjiang
37	JHX	15	Jilin
38	NMHNC	3	Inner Mongolia
39	HGLLC	3	Inner Mongolia
40	KZG	20	Inner Mongolia
41	HDH	30	Inner Mongolia
42	MLG	16	Inner Mongolia

43	DHB	9	Inner Mongolia
44	DSH	16	Inner Mongolia
45	LYT	6	Inner Mongolia
46	SJC	45	Ningxia
47	MGS	40	Ningxia

Supplementary Table 2. Summary of stem color variation among 873 AMM germplasm accessions from 47 natural populations

Stem color type	Number of accessions	Proportion (%)	Coefficient of variation (CV)
Red	110	12.6	
Green	495	56.7	
Variegated	268	30.7	
Total	873	100%	45.3

Supplementary Table 3. Overview of VOCs in the roots of AMM with different stem colors.

Compound	CAS	Formula	MW	RI	Rt (s)	Dt [a.u.]	Peak area	
							RR	GR
n-Nonanal	C124196	C9H18O	142.2	1101.9	372.759	1.48907	57.14±1.71	50.63±7.19
Furfuryl methyl sulfide	C1438911	C6H8OS	128.2	993.4	260.261	1.1387	86.57±2.75	76.15±6.8
6-Methyl-5-hepten-2-one	C110930	C8H14O	126.2	985.8	254.728	1.18075	52.63±19.92	30.09±3.3
Octamethylcyclotetrasiloxane	C556672	C8H24O4Si4	296.6	999.9	265.448	1.68861	71.22±5.33	128.65±3.4
2-Hexanol	C626937	C6H14O	102.2	794.1	150.846	1.28065	30.95±13.11	13.82±2.54
Isopentyl alcohol	C123513	C5H12O	88.1	734.4	121.825	1.24602	74.65±20.23	17.29±4.07
3-Pentanone	C96220	C5H10O	86.1	682.7	100.946	1.11257	231.04±20.58	219.1±31.56
1-Butanol	C71363	C4H10O	74.1	653	93.523	1.18286	71.85±21.19	48.24±10.17
2-Butanol(M)	C78922	C4H10O	74.1	626.1	87.259	1.15638	121.06±16.6	32.52±5.73
2-Butanol(D)	C78922	C4H10O	74.1	625	87.027	1.32242	86.3±46.35	19.1±2.32
2-Butanone(M)	C78933	C4H8O	72.1	587	78.908	1.05858	718.45±37.65	501.24±68.88
2-Butanone(D)	C78933	C4H8O	72.1	587	78.908	1.24398	612.26±140.01	125.18±65.84
Acetone	C67641	C3H6O	58.1	541.5	70.2	1.11482	233.95±30.8	93.78±39.54
1-Penten-3-ol	C616251	C5H10O	86.1	671.8	98.17	0.94099	82.69±7.55	39.72±13.73
Benzaldehyde	C100527	C7H6O	106.1	955.8	233.955	1.15004	27.29±2.39	21.63±2.47
Ethyl acetate	C141786	C4H8O2	88.1	601.5	81.913	1.09905	50.03±5.97	49.24±7.41
Butanal	C123728	C4H8O	72.1	596.5	80.864	1.28981	70.68±17.92	20.62±8.8
alpha-Pinene	C80568	C10H16	136.2	930.5	217.737	1.21905	18.72±4.62	12.18±1.15
p-Cymene	C99876	C10H14	134.2	1027.4	290.151	1.30793	47.87±9.9	19.09±1.73
Propylsulfide	C111477	C6H14S	118.2	893.9	196.319	1.15708	32.53±8.91	20.17±0.41
Methyl isobutyl ketone	C108101	C6H12O	100.2	729.9	119.747	1.17395	90.11±14.02	29.72±2.3

Supplementary Table 10. Hub genes in the turquoise module.

**ID**	**kME**	**ID**	**kME**	**ID**	**kME**
Am01G040820	0.936948	Am04G005020	0.855005	Am07G006990	0.831142
Am03G033510	0.960368	Am07G001680	0.977417	Am03G027800	0.952388
Am05G021550	0.930062	Am04G027700	0.88455	Am05G023290	0.843822
Am08G003790	0.852899	Am05G009050	0.82275	Am08G003810	0.858813
Am08G003850	0.857349	Am05G020270	0.929578	Am03G019520	0.860667
Am01G019920	0.961457	Am02G040100	0.955024	Am01G029920	0.906894
NewGene_27993	0.91547	Am02G033140	0.845367	Am01G022840	0.852697
Am05G037230	0.914352	Am02G040030	0.952865	Am03G024050	0.896595
Am02G038070	0.835531	Am07G001340	0.813767	Am01G039520	0.970389
Am07G002570	0.846332	Am09G024550	0.959995	Am06G011210	0.887022
Am02G033790	0.976926	Am03G007490	0.914127	Am03G024940	0.92275
Am09G000820	0.926972	Am08G003840	0.857504	Am05G010280	0.857192
Am01G038470	0.946134	NewGene_18765	0.920941	Am06G001920	0.958368
Am07G003010	0.87639	Am05G020350	0.944969	Am03G037550	0.925778
Am09G015340	0.937904	Am06G008040	0.942028	Am02G019420	0.867821
Am04G001550	0.930074	Am06G026200	0.833233	Am06G017670	0.883031
Am02G011100	0.917293	Am06G004750	0.954627	Am04G011140	0.867061

Supplementary Table 11. Reference protein sequences used in the phylogenetic analysis of anthocyanidin 3-*O*-glucosyltransferases and anthocyanidin 3-*O*-glucoside 2″ -*O*-xylosyltransferases.

Accession No.	Species	Gene ID	Enzyme Type
Q9LVW3	*Arabidopsis thaliana*	AtA3G2XYLT	Anthocyanidin 3-O-glucoside 2”-O-xylosyltransferase
A0A2R6QCI1	*Actinidia chinensis* var. *chinensis*	AcA3G2XYLT	
A0A6I9TDI7	*Sesamum indicum*	SiA3G2XYLT	
A0A6P6SCZ2	*Coffea arabica*	CaA3G2XYLT1	
A0A6P6SE19	*Coffea arabica*	CaA3G2XYLT2	
A0A9D5AJE6	*Pisum sativum*	PsA3G2XYLT	
A0A9Q0S9G0	*Salix viminalis*	SvA3G2XYLT1	
A0A9Q0U748	*Salix viminalis*	SvA3G2XYLT2	
A0A9R0IV07	*Spinacia oleracea*	SoA3G2XYLT	
A0AA49C3C2	*Gymnema sylvestre*	GsA3G2XYLT	
A0AAD6M650	*Populus alba* x *Populus* x *berolinensis*	PaA3G2XYLT1	
A0AAD6Q781	*Populus alba* x *Populus* x *berolinensis*	PaA3G2XYLT2	
A0AAJ6TBH4	*Populus euphratica*	PeA3G2XYLT	
A0AAW2Q4M6	*Sesamum calycinum*	ScA3G2XYLT	
A0AAW2WPT7	*Sesamum latifolium*	SlA3G2XYLT	

A0ABM0X9U6	*Camelina sativa*	CsA3G2XYLT1	
A0ACB8NHA7	*Citrus sinensis*	CsA3G2XYLT2	
P51094	*Vitis vinifera*	VvUFGT	Anthocyanidin 3-O-glucosyltransferase
Q66PF5	*Fragaria ananassa*	FaUFGT1	
Q9LFJ8	*Arabidopsis thaliana*	AtUFGT	
Q5UL10	*Fragaria ananassa*	FaUFGT2	
P14726	*Hordeum vulgare*	HvUFGT	
P16165	*Zea mays*	ZmUFGT1	
P16166	*Zea mays*	ZmUFGT2	
P16167	*Zea mays*	ZmUFGT3	
Q40284	*Manihot esculenta*	MeUFGT1	
Q40287	*Manihot esculenta*	MeUFGT2	
Q43641	*Solanum melongena*	SmUFGT	
Q96493	*Gentiana triflora*	GtUFGT	

Supplementary Table 12. Primer sequences used for qRT-PCR analysis.

Gene name	Primer	Sequence (5'- 3')
AmF3'H	Forward primer	GACCACCCAACGCTGGAG
	Reverse primer	GGCCTTGCCGGAGAAGAG
AmBZ1	Forward primer	TGGGTTGGTGGAAAGCTTGA
	Reverse primer	GGCCCAGCTGCATCTACC
AmCHI	Forward primer	TCCTCCGCCAACACCCTA
	Reverse primer	GCAACAGCGTTGTCCTGC
AmF3H	Forward primer	TGGCACCAGCGAGAACTC
	Reverse primer	GCGAGGGAAATGACAGGGA
AmCHS	Forward primer	CAGAAGGCCCTGCAACCA
	Reverse primer	TCGGTCTTGTGCTCGCTG
AmC4H	Forward primer	CCGGTGCCAATCTTCGGT
	Reverse primer	TCCCATGCGGAGGAGGAA
18S	Forward primer	TGCAGAATCCCGTGAACCATC
	Reverse primer	AGGCATCGGGCAACGATATG

Supplementary Table 13. Primer sequences used for amplification of six stem color-related biosynthetic genes in AMM.

Gene name	Primer	Sequence (5'- 3')
AmF3'H	Forward primer	CAAGGAGAAAAAACCCCGGATCCATGTCTCCATGGCTTATTTCCTT
	Reverse primer	CCCTATAGTGAGTCGTATTACTCACACAGATGATGAGTACACAT
AmBZ1	Forward primer	CAGCAAATGGGTCGCGGATCCATGACAGAGAACAAACATGTAGCAGTC
	Reverse primer	CTCGAGTGCGGCCGCAAGCTTTTAAGAGCTAGAAGAAACTAGTTCCACC
AmCHI	Forward primer	CAGCAAATGGGTCGCGGATCCATGGCACCGCCGATCGTC
	Reverse primer	CTCGAGTGCGGCCGCAAGCTTTCAGTTAACAGTCTCACCACCCTG
AmF3H	Forward primer	CAGCAAATGGGTCGCGGATCCATGGCACCAGCGAGAACTCTC
	Reverse primer	CTCGAGTGCGGCCGCAAGCTTCTAAGCAAGAATCTCATTCAAAGGTT
AmCHS	Forward primer	CAGCAAATGGGTCGCGGATCCATGGTGAGTGTTGCTGAAATTCG

	Reverse primer	CTCGAGTGCGGCCGCAAGCTTTTAGATAGCCACACTACGCAGAACA
AmC4H	Forward primer	CAAGGAGAAAAAACCCCGGATCCATGGATCTCCTCCTCTTAGAGAA
	Reverse primer	CCCTATAGTGAGTCGTATTACCTAAAATGATCTTGGCTTTAAGA
